# Open Safety Pin Ingestion Presenting as Incarcerated Umbilical Hernia

**Published:** 2011-11-27

**Authors:** Bilal Mirza, Afzal Sheikh

**Affiliations:** Department of Pediatric Surgery, The Children's Hospital and the Institute of Child Health Lahore, Pakistan

**Keywords:** Safety pin ingestion, Strangulated umbilical hernia, Intestinal perforation

## Abstract

Foreign body ingestion is common in children. Sharp foreign bodies are potentially harmful and can result various complications. An 8-month-old infant presented with incarcerated umbilical hernia. With a suspicion of strangulation, operation was performed that revealed a loop of ileum being stuck in the umbilical defect. The loop of ileum was freed from the umbilicus which demonstrated open ends of safety pin piercing out of bowel lumen. The enterotomy followed by removal of safety pin was performed.

## INTRODUCTION

Sharp foreign bodies in alimentary tract can produce a number of complications that often need surgery. The diagnosis is often overt while dealing with the complications. The presentation of safety pin as incarcerated inguinal hernia is seldom reported, however, its presentation as incarcerated umbilical hernia is never described in English literature [1, 2, 3, 4]. We are reporting a case of open safety pin ingestion presenting with incarcerated umbilical hernia.

## CASE REPORT

An 8-month-old male infant presented with incarcerated umbilical hernia. The umbilical hernia was initially reducible but for a day it turned irreducible. General physical examination was unremarkable (Temp 99F, Pulse 90/min, respiration rate 25/min, BP within normal limits). Abdominal examination showed abdominal tenderness around umbilicus and an irreducible umbilical hernia. Laboratory investigations were normal. Ultrasound of the abdomen pointed a loop of bowel being stuck in the umbilicus. X ray abdomen was not performed.

Operation was performed by making semi-lunar infra-umbilical incision that revealed a loop of mid ileum entrapped at the umbilicus. The loop of ileum was reduced. Further exploration revealed an open safety pin causing perforation of the ileum (Fig. 1). The safety pin was removed by an enterotomy at the site of perforation. The enterotomy was then closed. Umbilical hernia was also repaired. The postoperative recovery was uneventful.

**Figure:**
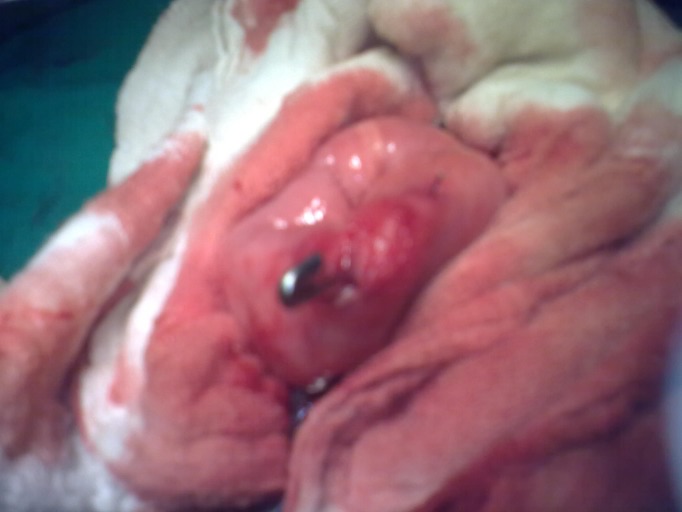
Figure 1: showing an open safety pin perforating the ileum.

## DISCUSSION

Various sharp FBs that are ingested include safety pins, sewing needles, hair pin, nails etc. In many cases the sharp FB cause complications. The reported complications are esophageal perforation leading to mediastinitis, pneumothorax, pneumomediastinum, secondary tracheoesophageal fistula, gastric perforation, perforation of small and large bowel leading to peritonitis, heart perforation, duodenocolic fistula, incarcerated inguinal hernia, and the like [1, 2, 3, 4]. Rarely, ingested sharp FB presents with incarcerated inguinal hernia [4]. In our case the patient developed incarcerated umbilical hernia due to inflammation and adhesions resulting from the small bowel perforation by open safety pin. The presentation of ingested safety pin as incarcerated umbilical hernia is a rare event and not reported before.

## Footnotes

**Source of Support:** Nil

**Conflict of Interest:** None declared
